# Gene expression profiles classifying clinical stages of tuberculosis and monitoring treatment responses in Ethiopian HIV-negative and HIV-positive cohorts

**DOI:** 10.1371/journal.pone.0226137

**Published:** 2019-12-10

**Authors:** Gebremedhin Gebremicael, Desta Kassa, Yodit Alemayehu, Atsbeha Gebreegziaxier, Yonas Kassahun, Debbie van Baarle, Tom H. M. Ottenhoff, Jacqueline M. Cliff, Mariëlle C. Haks

**Affiliations:** 1 HIV and TB Diseases Research Directorate, Ethiopian Public Health Institute (EPHI), Addis Ababa, Ethiopia; 2 TB Centre and Department of Immunology and Infection, Faculty of Infectious and Tropical Diseases, London School of Hygiene & Tropical Medicine, London, England, United Kingdom; 3 Armauer Hansen Research Institute, Addis Ababa, Ethiopia; 4 Center for Immunology of Infectious Diseases and Vaccins (IIV), National Institute for Public Health and the Environment (RIVM), Bilthoven, The Netherlands; 5 Department of Infectious Diseases, Leiden University Medical Center, Leiden, The Netherlands; University of Arizona College of Medicine, UNITED STATES

## Abstract

**Background:**

Validation of previously identified candidate biomarkers and identification of additional candidate gene expression profiles to facilitate diagnosis of tuberculosis (TB) disease and monitoring treatment responses in the Ethiopian context is vital for improving TB control in the future.

**Methods:**

Expression levels of 105 immune-related genes were determined in the blood of 80 HIV-negative study participants composed of 40 active TB cases, 20 latent TB infected individuals with positive tuberculin skin test (TST+), and 20 healthy controls with no *Mycobacterium tuberculosis* (*Mtb*) infection (TST-), using focused gene expression profiling by dual-color Reverse-Transcription Multiplex Ligation-dependent Probe Amplification assay. Gene expression levels were also measured six months after anti-TB treatment (ATT) and follow-up in 38 TB patients.

**Results:**

The expression of 15 host genes in TB patients could accurately discriminate between TB cases versus both TST+ and TST- controls at baseline and thus holds promise as biomarker signature to classify active TB disease versus latent TB infection in an Ethiopian setting. Interestingly, the expression levels of most genes that markedly discriminated between TB cases versus TST+ or TST- controls did not normalize following completion of ATT therapy at 6 months (except for *PTPRCv1*, *FCGR1A*, *GZMB*, *CASP8* and *GNLY*) but had only fully normalized at the 18 months follow-up time point. Of note, network analysis comparing TB-associated host genes identified in the current HIV-negative TB cohort to TB-associated genes identified in our previously published Ethiopian HIV-positive TB cohort, revealed an over-representation of pattern recognition receptors including *TLR2* and *TLR4* in the HIV-positive cohort which was not seen in the HIV-negative cohort. Moreover, using ROC cutoff ≥ 0.80, *FCGR1A* was the only marker with classifying potential between TB infection and TB disease regardless of HIV status.

**Conclusions:**

Our data indicate that complex gene expression signatures are required to measure blood transcriptomic responses during and after successful ATT to fully diagnose TB disease and characterise drug-induced relapse-free cure, combining genes which resolve completely during the 6-months treatment phase of therapy with genes that only fully return to normal levels during the post-treatment resolution phase.

## Background

Tuberculosis (TB) is a leading cause of death [1] and 25% of the 10.0 million incident TB disease cases globally were reported in Africa during 2017 [2]. WHO recommends developing effective diagnostic tests and treatments for latent TB infection (LTBI) to achieve a 90% and 80% reduction of the incidence and death rate from *Mycobacterium tuberculosis* (*Mtb*) respectively by 2030 [3]. The currently available diagnostic tools (smear microscopy, solid and liquid sputum culture, Genexpert) have several limitations to detect latent and active TB [4,5,6,7] and for monitoring TB treatment response [8], and those limitations greatly contribute to the spread of TB disease.

Because existing immunological methods to diagnose TB infection, such as the tuberculin skin test (TST) and Interferon-γ release assays (IGRAs), are not able to distinguish between LTBI and active TB disease [9], it has been suggested that the identification of biomarkers that can classify clinical stages of TB and monitor TB treatment responses is essential and cost-effective for improving clinical practice [10]. Changes in gene expression in peripheral blood due to the interaction between the host immune response and *Mtb* could potentially be used as biomarkers to classify the different clinical outcomes of TB exposure and to monitor TB treatment response. There have been previous studies showing that various stages of *Mtb* infection can be distinguished using gene expression profiling in peripheral blood for the diagnosis of TB disease and monitoring TB treatment [11,12,13,14,15,16,17,18] in cohorts from Europe, North and South America, Asia and Africa (South Africa, Malawi and Gambia). For instance, Wu and colleagues [15] identified 10 genes whose expression differentiated patients with active TB disease from LTBI individuals in a North American cohort. Kaforou and colleagues [16] identified and validated a 44 gene signature that distinguished active tuberculosis from other diseases in different African cohorts, while Warsinske and his colleagues [17] identified a 3-gene messenger RNA expression score that distinguished individuals who progressed to TB cases from non progressors, individuals with TB cases from non TB cases, and individuals with slower treatment response during TB therapy in Brazil and South Africa. However, those host markers may not be applicable in another population, because various studies have indicated that diverse genetic backgrounds and environmental factors impact on gene expression and cytokine profiles in peripheral blood [19,20]. Mihret and colleagues [21] found 9 host genes, identified from a limited panel of 45 host genes, which discriminated patients with active TB disease from household contacts in the context of Ethiopia. However, validating those signatures and identifying additional candidate genes for diagnosing TB disease will be important. Therefore, in this study we aimed to validate and identify novel candidate host gene biomarkers that classify active TB disease and that can be used to monitor TB treatment responses in the context of Ethiopia, using focused gene expression profiling by dual-color Reverse-Transcription Multiplex Ligation-dependent Probe Amplification (dcRT-MLPA).

## Materials and methods

### Ethics statement

All study participants provided written, informed consent at enrollment. The study obtained ethical clearance from the Scientific and Ethics Research Office (Ref: EHNRI 6.13/01), the Ethiopian Public Health Research Institute, and the London School of Hygiene & Tropical Medicine Ethics Review Committee (Ref:7174).

### Study design and population

An observational cohort study was conducted between April 2007 and January 2011 at three health facilities (St. Peter Specialized TB Hospital, Akaki and Kality Health Centers), Addis Ababa, Ethiopia. Study participants were adults of both sexes ranging between 15–65 years of age. Demographic data of the study participants were collected using a standard questionnaire at recruitment and follow-up. A total of 80 study participants were enrolled, including 40 active TB cases, 20 latent TB infected individuals (tuberculin skin test positive; TST+), and 20 healthy controls (TST-) and they were all HIV negative. The latent TB and control group (20 TST+ and 20 TST- subjects) had no prior diagnosis of TB and were recruited without any clinical symptoms or signs of illness due to active TB and HIV/AIDS. Possible study participants who refused HIV testing, were pregnant, had co-morbidity with diabetes mellitus or chronic bronchitis, were receiving steroid therapy, had received TB treatment (at recruitment or previously), or who had alcohol or drug abuse that could compromise reliability, were excluded from the enrollment. All active TB cases confirmed at enrollment were treated according to the national guideline [22] and followed until the end of anti-tuberculosis treatment (ATT) at 6 months (6M) and additionally at 18 months (18M). Furthermore, they were negative for Acid Fast Bacilli (AFB) by microscopy and clinically asymptomatic at 6M and 18M.

### Diagnostic assessment

The HIV status of study participants was determined using the Determine HIV-½ (Abbott laboratories, Japan) as the screening test, the Capilus HIV-½ (TrinityBiotec, Ireland) as the confirmatory test and Unigold HIV-½ recombinant (TrinityBiotec, Ireland) as a tie breaker test [22]. The CD4 count was determined by flow cytometry using a FACSCalibur Flow cytometer (Becton Dickinson, San Jos, USA).

Active TB diagnosis was based on both clinical and bacteriological parameters. At least two sputum smears (“spot-early morning”) were required to be positive by microscopy for Acid Fast Bacilli (AFB) using the Ziehl-Neelsen staining method [22]. A TST test to detect latent TB infection was performed at baseline and follow-up visits for all participants except active TB patients according to the national guidelines [22]. A 0.1ml tuberculin solution (RT23, State Serum Institute, Copenhagen) was injected intradermally into the dorsal surface of the forearm. TST positivity was classified as skin induration diameter ≥10 mm in HIV-uninfected individuals [22].

### RNA extraction

RNA was extracted from 2.5ml blood collected in Paxgene tubes (PreAnalytiX, Qiagen, Germany) using the Paxgene RNA extraction kit (PreAnalytiX, Qiagen) according to the manufacturer’s instructions. Briefly, Paxgene tubes were centrifuged at 4000 rpm for 10 minutes and the pellet was lysed and resuspended by Resuspension Buffer (Buffer BR1), followed by treatment with proteinase K to remove contaminating proteins. Ethanol-precipitated nucleic acids were loaded onto a spin column followed by on-column DNA digestion using RNase-free DNase (Qiagen). Finally, purified RNA was eluted with RNase-free buffer (BR5 buffer) and quantified using a NanoDrop 2000 Spectrophotometer (Thermo Fisher Scientific, Wilmington, USA). RNA samples with 260/280 nm absorbance ratios below 1.70 or above 2.3 were excluded from further analyses.

### Dual-color Reverse-Transcription Multiplex Ligation-dependent Probe Amplification (dcRT-MLPA)

DcRT-MLPA was performed as described in detail elsewhere [18]. Briefly, for each target‐specific sequence, a specific reverse transcription (RT) primer was designed located immediately downstream of the left and right-hand half‐probe target sequence. Complementary DNA (cDNA) was generated from RNA using an RT primer mix. Subsequently, MMLV reverse transcriptase was inactivated by heating at 98°C for 2 minutes and cDNA was incubated overnight at 60°C with a mixture of customized left and right-hand half‐probes to hybridize with the target cDNA. Annealed half‐probes were ligated using ligase-65 enzyme and subsequently amplified by PCR (33 cycles of 30 sec at 95°C, 30 sec at 58°C, and 60 sec at 72°C, followed by 1 cycle of 20 min at 72°C). Primers and probes were from Sigma-Aldrich Chemie (Zwijndrecht, The Netherlands) and MLPA reagents from MRC-Holland (Amsterdam, The Netherlands). PCR amplification products were 1:10 diluted in HiDi formamide‐containing 400HD ROX size standard, denatured at 95^ o^C for 5 min, cooled on ice and analyzed on an Applied Biosystems 3730 capillary sequencer in GeneScan mode (Base Clear, Leiden, The Netherlands).

Trace data were analyzed using GeneMapper software 5 package (Applied Biosystems). The areas of each assigned peak (in arbitrary units) were exported for further analysis in Microsoft Excel spreadsheet software. Data were normalized to GAPDH and signals below the threshold value for noise cutoff in GeneMapper (log2 transformed peak area 7.64) were assigned the threshold value for noise cutoff. Finally, the normalized data were log2 transformed for statistical analysis.

RT primers and half-probes were designed by Leiden University Medical Centre (LUMC, Leiden, The Netherlands) [18,23] and comprised sequences for 4 housekeeping genes and 105 selected genes to profile the innate and adaptive immune response (**S1 Table**). Genes associated with active TB disease or protection against disease, as described in the literature, were included in the study.

### Statistical analysis

The Kolmogorov Smirnov test showed the data were not normally distributed. A non-parametric Kruskal-Wallis H test was used to compare medians among more than two clinical groups. A non-parametric two tailed Wilcoxon rank-sum (Mann-Whitney) test was used to compare two unpaired data sets while a Wilcoxon signed-rank test was used for two paired data sets., Ingenuity Pathway Analysis (IPA) was used to look the network of those genes that discriminate TB cases from controls in HIV positive and HIV negative patients. The statistical significance level used was P<0.05 and all *P* values are two-tailed. All data analysis was performed using Inter cooled STATA version 11.0 (College Station, Texas, USA).

## Results

### Characteristics of the study population

A total of 80 HIV-negative study participants composed of 40 TB cases, 20 TST+ and 20 TST- were included in this study. Malnutrition (BMI<18.5 kg/m2) was detected in 52% of TB patients compared to 15% of TST+ and 0% of TST- individuals (**Table 1**).

**Table 1:** Baseline demographic and clinical characteristics of the study populations.

Characteristics	TB cases (n = 40)	TST+ (n = 20	TST- (n = 20)	P-value
**Demographic data**				
Age, years	27 ± 9.3	23 ± 6.3	22.5 ± 6.0	0.0546
Female, n (%)	17 (42.5)	14 (70)	14 (70)	**0.046**
Median of BMI, kg/m^2^ (IQR)	18.4 (16.9–20.0)	21.6 (19.2–23.5)	21.3 (19.5–23.1)	**0.0001**
**Nutritional status**				
Severe malnutrition, n (%)	6 (15)	0 (0)	0 (0)	
Moderate malnutrition, n (%)	4 (10)	0 (0)	0 (0)	
Mild malnutrition, n (%)	11 (27.5)	3 (15)	0 (0)	
Normal, n (%)	17 (42.5)	14 (70)	18 (90)	
Overweight, n (%)	2 (5)	3 (15)	2 (10)	
CD4^±^ T cell count				
Median CD4^+^ T cell count (IQR)	426 (292.5–636)	713.5 (573.5–943.5)	821 (693–903)	**0.0001**
CD4^+^ T cell count ≤ 200 cells/ μl, n (%)	16 (40)	1 (5)	1 (5)	0.1048

Data indicate medians ± standard deviations unless stated otherwise. BMI cutoff of <18.5 kg/m^2^ was used to define underweight. A CD4^+^ T-cell count cutoff of <200 cells/μl was used to define lymphocytopenia. n (%): Number of patients (Percentage of patients); BMI: Body Mass Index. P-values ≤ 0.05 are indicated in bold.

### Gene expression profiles descriminating active TB from latent infection

Whole blood gene expression levels of TB cases, TST+ and TST- individuals were analyzed by dcRT-MLPA using probe sets for 105 selected genes to profile innate and adaptive immune responses (**S1 Table**). Of the 105 host genes analysed, 54 genes were not differentially expressed between the three clinical groups (TB cases, TST+ and TST-) and were excluded from further analysis. Thirty nine genes, including *CD19*, *NCAM1*, *CD3E*, *CD4*, *CD8A*, *CCR7*, *IL7R*, *PTPRCv1*, *IL2*, *GATA3*, *IL5*, *IL13*, *CCL4*, *CTLA4*, *GNLY*, *GZMB*, *PRF1*, *CASP8*, *BCL2*, *TNFRSF1A*, *TNFRSF1B*, *CD163*, *CCL5*, *CCL22*, *CXCL13*, *IL12B*, *TLR9*, *NLRP1*, *NLRP2*, *NLRP12*, *NLRP13*, *TIMP2*, *AREG*, *TGFBR2*, *RAB33A*, *BPI*, *TWIST1*, *SEC14L1*, and *BLR1*, had significantly lower expression in TB cases compared to TST+ subjects, while 9 genes including AIRE, CCL2, IL23A, MRC2, NOD2, TLR3, TLR5, FCGR1A and TAGAP were significantly more highly expressed in TB patients compared to TST+ subjects (**Table 2**).

**Table 2:** Gene expression profiles differentiating between study groups at baseline (0M).

Gene Symbol	TB cases (n = 40)	TST+ (n = 20)	TST- (n = 20)	TB cases vs TST+ P-value	TB cases vs TST- P-value	TST+ vs TST- P-value
**Immune cell subset markers**						
CD19	7.6(7.6–7.6)	7.7(7.6–8.1)	8.2(7.8–8.6)	**0.0000**	**0.0000**	**0.0424**
NCAM1	8.4(7.6–8.7)	9.4(8.8–9.9)	9.3(8.7–9.6)	**0.0000**	**0.0002**	0.6456
**T cell subset markers**						
CD3E	12.5(11.3–13.0)	14.2(13.8–14.6)	14.1(13.7–14.3)	**0.0000**	**0.0000**	0.3577
CD4	12.0(11.4–12.5)	12.8(12.4–13.3)	12.3(12.1–12.9)	**0.0001**	**0.0137**	0.0834
CD8A	12.7(12.3–13.0)	13.0(12.8–13.4)	13.1(12.9–13.2)	**0.0021**	**0.0005**	1.0000
CCR7	12.8(12.2–13.4)	14.7(14.1–15.0)	14.2(14.0–14.4)	**0.0000**	**0.0000**	0.0620
IL7R	12.1(11.7–13.0)	14.1(13.7–14.5)	14.1(13.8–14.4)	**0.0000**	**0.0000**	0.8077
PTPRCv1	10.7(10.2–11.3)	12.1(11.5–12.3)	12.0(11.3–12.2)	**0.0000**	**0.0000**	0.5518
AIRE	11.9(7.6–12.9)	7.6(7.6–7.6)	7.6(7.6–7.6)	**0.0027**	**0.0027**	1.0000
**Th1/2/9/17 associated genes/Treg associated genes**						
IL2	9.1(8.4–9.6)	10.0(9.4–10.7)	9.4(9.1–9.7)	**0.0014**	0.0755	**0.0373**
TNF	10.8(9.7–11.5)	10.2(10.0–10.5)	9.9(9.7–10.0)	0.2298	**0.0163**	**0.0005**
GATA3	7.6(7.6–7.6)	7.7(7.6–8.0)	7.8(7.6–8.3)	**0.0000**	**0.0000**	0.7568
IL5	7.6(7.6–12.1)	14.3(14.0–14.9)	13.6(13.4–13.8)	**0.0000**	**0.0000**	**0.0002**
IL13	7.6(7.6–7.6)	10.3(7.6–11.3)	8.9(7.6–10.3)	**0.0001**	**0.0022**	0.1755
CCL4	9.5(9.0–10.0)	10.1(10.0–10.3)	10.1(9.8–10.3)	**0.0003**	**0.0051**	0.4652
CTLA4	12.0(11.5–12.4)	12.6(12.2–12.7)	12.4(12.1–12.7)	**0.0025**	**0.0091**	0.4652
**Cytotoxicity genes**						
GNLY	13.4(12.9–14.2)	15.0(14.4–15.4)	14.6(14.3–15.0)	**0.0000**	**0.0001**	0.1595
GZMB	11.7(11.2–12.3)	12.6(12.0–13.0)	12.7(12.2–12.9)	**0.0009**	**0.0010**	0.8711
PRF1	7.6(7.6–12.3)	13.9(13.2–14.2)	13.8(13.2–14.2)	**0.0000**	**0.0000**	0.8287
**Apoptosis/survival**						
CASP8	12.3(11.9–12.7)	12.8(12.6–13.0)	12.9(12.5–13.1)	**0.0002**	**0.0010**	1.0000
BCL2	9.5(8.5–9.9)	10.6(10.1–10.9)	10.2(10.0–11.0)	**0.0000**	**0.0000**	0.4171
TNFRSF1A	13.6(13.1–13.8)	13.9(13.7–14.2)	13.8(13.5–14.0)	**0.0026**	**0.0374**	0.2235
TNFRSF1B	11.4(10.8–11.8)	11.9(11.7–12.2)	11.5(11.3–11.9)	**0.0014**	0.2869	**0.0094**
**Myeloid associated genes**						
CD163	8.9(8.6–9.3)	9.3(9.0–9.6)	9.4(9.3–9.7)	**0.0029**	**0.0000**	0.2235
CCL2	7.9(7.6–9.4)	7.6(7.6–8.2)	7.6(7.6–7.6)	**0.0410**	**0.0103**	0.3188
CCL5	13.9(13.6–14.1)	14.6(14.3–15.0)	14.5(14.3–14.9)	**0.0000**	**0.0002**	0.3577
CCL22	13.9(12.3–15.3)	14.8(14.4–15.5)	13.9(13.8–14.2)	**0.0178**	0.8854	**0.0003**
CXCL13	10.8(10.2–11.2)	11.3(11.0–11.4)	11.1(10.7–11.3)	**0.0010**	0.0837	0.1298
IL12B	7.6(7.6–8.2)	8.6(8.3–9.1)	7.9(7.6–8.6)	**0.0000**	0.0524	**0.0055**
IL23A	11.4(11.3–11.7)	9.5(8.4–11.3)	11.3(11.3–11.4)	**0.0000**	0.0547	**0.0009**
**Pattern recognition receptors**						
MRC2	7.6(7.6–8.8)	7.6(7.6–7.6)	7.6(7.6–7.6)	**0.0007**	**0.0007**	1.0000
NOD2	8.9(8.0–9.5)	8.0(7.6–8.4)	7.7(7.6–8.0)	**0.0004**	**0.0000**	0.3677
TLR3	10.1(9.5–10.8)	9.4(9.3–9.8)	10.5(9.8–10.9)	**0.0149**	0.4760	**0.0080**
TLR5	13.6(8.8–15.1)	7.6(7.6–8.0)	8.4(7.6–10.7)	**0.0000**	**0.0001**	**0.0302**
TLR9	11.8(7.6–14.2)	15.5(15.1–15.9)	14.9(14.9–15.3)	**0.0000**	**0.0000**	**0.0063**
**Inflammasome components**						
NLRP1	7.6(7.6–9.5)	11.1(10.8–11.6)	11.1(10.7–11.5)	**0.0000**	**0.0000**	0.7455
NLRP2	11.1(10.0–12.0)	12.7(11.9–13.4)	11.7(11.6–12.1)	**0.0000**	**0.0082**	**0.0102**
NLRP12	8.4(8.0–8.7)	9.2(9.1–9.5)	9.3(8.9–9.5)	**0.0000**	**0.0000**	0.6652
NLRP13	7.6(7.6–8.9)	8.6(8.1–9.8)	8.1(7.6–8.9)	**0.0087**	0.4112	**0.0490**
**IFN signalling genes**						
FCGR1A	11.4(10.7–11.7)	9.4(9.1–10.4)	8.8(8.1–9.3)	**0.0000**	**0.0000**	**0.0014**
**Inflammation**						
TIMP2	14.3(13.5–14.7)	14.6(14.4–14.8)	14.5(14.2–14.7)	**0.0170**	0.2623	0.1850
**Other**						
AREG	7.6(7.6–12.1)	11.9(11.8–12.3)	12.0(11.5–12.4)	**0.0005**	**0.0004**	0.8498
TGFBR2	11.5(11.0–12.0)	11.9(11.7–12.3)	11.7(11.4–12.1)	**0.0156**	0.3130	0.1595
RAB13	8.2(7.6–8.8)	9.0(7.6–10.0)	9.4(8.4–9.8)	0.0860	**0.0011**	0.7128
RAB24	11.5(11.1–11.8)	11.2(10.8–11.5)	10.9(10.6–11.1)	0.1735	**0.0008**	**0.0305**
RAB33A	7.6(7.6–7.6)	8.3(7.8–8.8)	8.2(7.7–8.6)	**0.0000**	**0.0000**	0.6832
TAGAP	12.6(12.1–13.0)	13.5(13.3–13.5)	13.4(13.2–13.6)	**0.0000**	**0.0000**	0.7251
BPI	14.5(13.7–15.0)	15.2(14.9–15.4)	14.6(14.5–14.9)	**0.0001**	0.0704	**0.0029**
TWIST1	7.6(7.6–7.6)	7.6(7.6–8.0)	7.6(7.6–7.6)	**0.0030**	0.4739	**0.0090**
SEC14L1	13.9(13.7–14.3)	14.3(14.1–14.9)	14.2(13.9–14.8)	**0.0143**	**0.0420**	0.5162
BLR1	9.4(9.2–10.1)	10.8(10.3–11.1)	10.8(10.5–11.4)	**0.0000**	**0.0000**	0.4989

Median (inter quartile range) gene expression values (peak areas normalized for GAPDH and log2-transformed) are shown at baseline and significant differences between study groups were determined using Kruskal-Wallis H and Wilcoxon Mann-Whitney test. In red, genes are indicated that were more highly expressed in the test group compared to the reference/control group while blue indicates genes that had lower expression in the test group compared to the reference/control group. Only genes whose expression level significantly differed between any of the study groups are listed. P-values ≤ 0.05 are indicated in bold.

**Fig 1:**
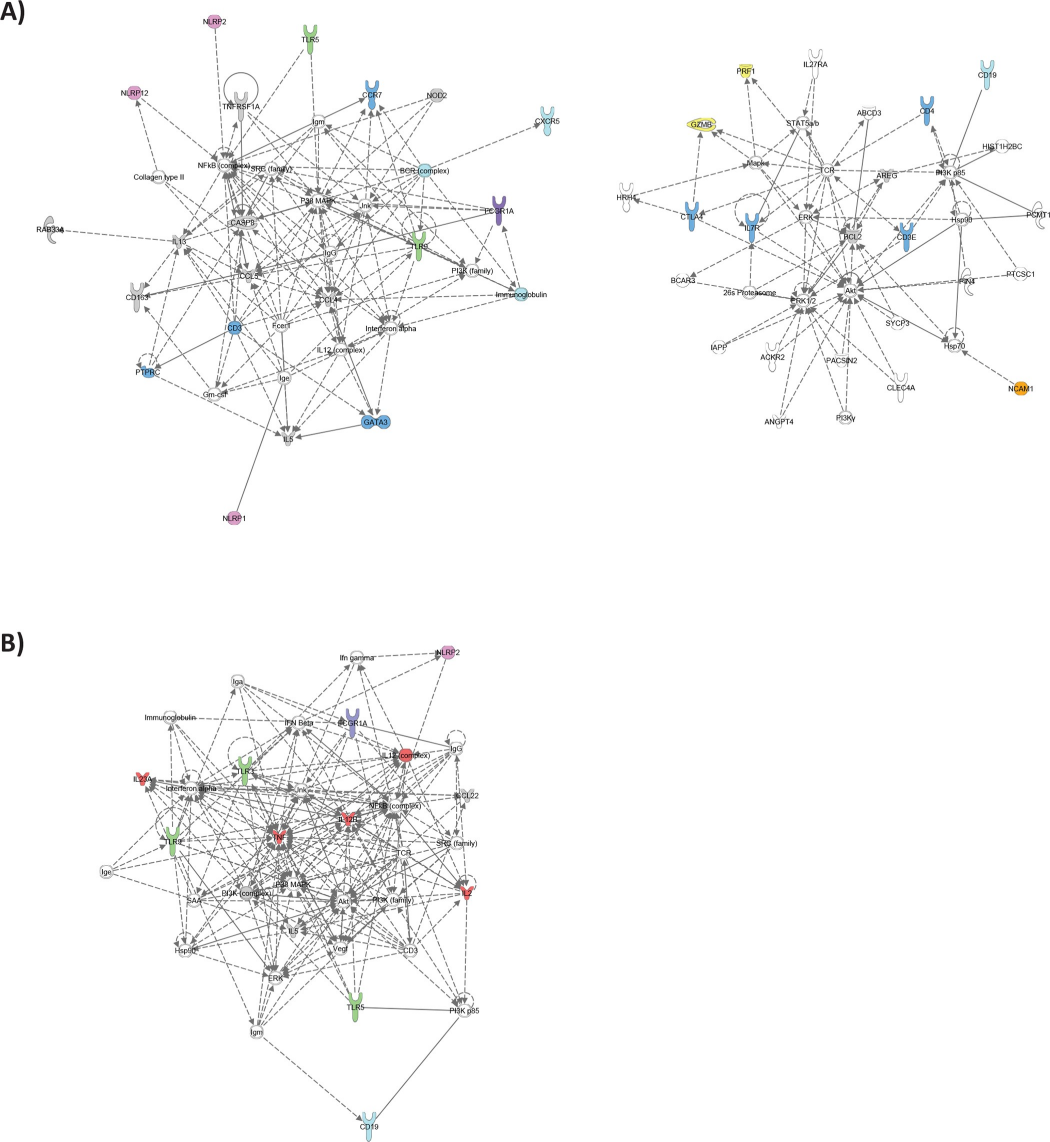
IPA network analysis. Ingenuity Pathway Analysis in HIV-negative individuals of (A) TB-associated genes that were differentially expressed between TB cases and TST+ individuals as well as between TB cases and TST- individuals at baseline and (B) genes that were differentially expressed between TST+ and TST- individuals at baseline. Dark blue: T cell associated genes, Light blue: B cell associated genes, Yellow: Cytotoxicity associated genes, Green: Pattern recognition receptors, Purple: IFN-inducible genes, Pink: Inflammasome components, Orange: NK cell associated genes, Red: Pro-inflammatory cytokines.

Thirty-one host genes including CD19, NCAM1, CD3E, CD4, CD8A, CCR7, IL7R, PTPRCv1, GATA3, IL5, IL13, CCL4, CTLA4, GNLY, GZMB, PRF1, CASP8, BCL2, TNFRSF1A, CD163, CCL2, CCL5, TLR9, NLRP1, NLRP2, NLRP12, AREG, RAB13, RAB33A, SEC14L1 and BLR1 had significantly lower expression in TB patients compared to TST- subjects; while 8 genes including AIRE, TNF, MRC2, NOD2, TLR5, FCGR1A, RAB24 and TAGAP were significantly more highly expressed in TB cases compared to TST- subjects. All except 4 host genes (TNF, CCL2, RAB13 and RAB24) that were differentially expressed between TB cases and TST- also discriminated between TB cases and TST+ (**Table 2**), suggesting that these biomarkers might be strongly associated with TB disease. Ingenuity pathway network analysis was performed to identify regulatory networks and key genes and biological pathways: it indicated that the TB associated signature primarily consisted of two networks of genes identifying immune cell subsets, inflammasome components, pattern recognition receptors and cytotoxicity genes (**Fig 1A**).

Of the 17 genes that were differentially expressed between TST+ and TST-, 4 genes, including CD19, IL23A, TLR3 and TLR5, had significantly lower expression in TST+ compared to TST-, whereas 13 genes including IL2, TNF, IL5, TNFRSF1B, CCL22, IL12B, TLR9, NLRP2, NLRP13, FCGR1A, RAB24, BPI and TWIST1, had significantly higher expression (**Table 2**). IPA analysis of these differences between TST+ and TST- subjects identified a network centered around pro-inflammatory cytokines and containing multiple pattern recognition receptors (**Fig 1B**).

**Fig 2:**
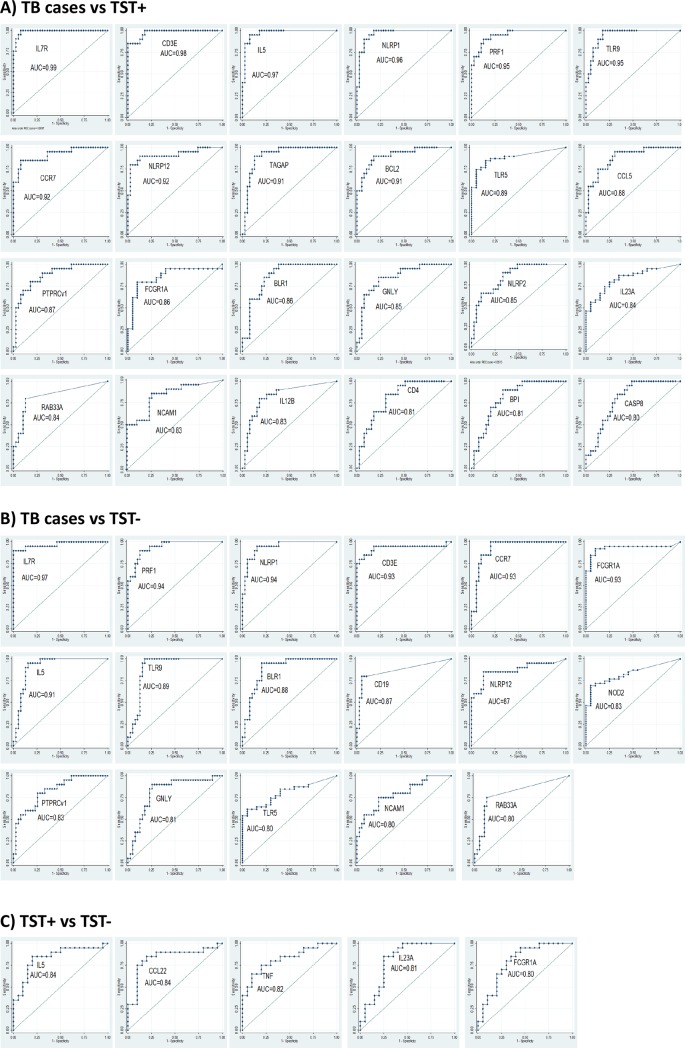
Identification of single genes with discriminatory power to classify HIV-negative study groups at baseline (M0). Receiver operator characteristics (ROC) curves showing the accuracies of individual genes in discriminating (A) TB cases versus TST+ subjects, (B) TB cases versus TST- subjects and (C) TST+ versus TST- subjects. AUC = Area under the curve.

**Fig 3:**
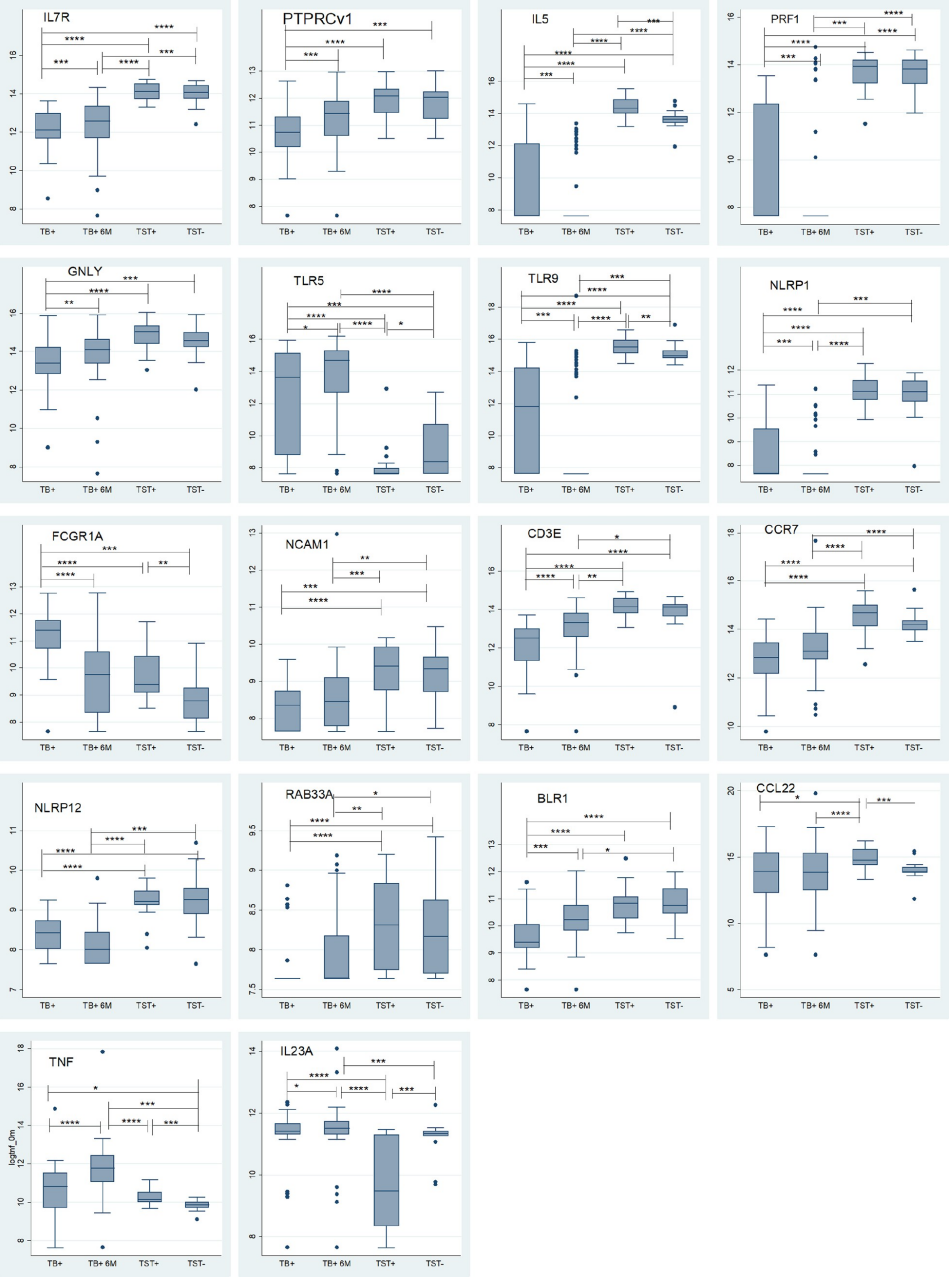
Gene expression profiles of signature genes. Median gene expression levels (peak areas normalized to GAPDH and log2-transformed) of the indicated genes are shown as box-and-whisker plots (5–95 percentiles). Significant differences among the groups and between study groups were determined using Kruskal-Wallis H test and Wilcoxon Mann-Whitney test respectively. Shown are individual genes that were found to have the best discriminatory power (AUCs ≥ 0.80) to distinguish between active TB cases (TB) versus latently infected (TST+) and uninfected (TST-) controls in HIV-negative subjects. (* = P-value ≤0.05, ** = P-value ≤0.01, *** = P-value ≤0.001, **** = P-value ≤0.0001).

Non-parametric Receiver Operator Characteristic (ROC) curves to determine the discriminatory potential of single genes identified IL7R, CD3E, IL5, NLRP1, PRF1, TLR9, CCR7, NLRP12, TAGAP, BCL2, TLR5, CCL5, PTPRCv1, FCGR1A, BLR1, GNLY, NLRP2, IL23A, RAB33A, NCAM1, IL12B, CD4, BPI and CASP8 with Area Under the Curve (AUCs) of 0.99, 0.98, 0.97, 0.96, 0.95, 0.95, 0.92, 0.92, 0.91, 0.91, 0.89, 0.88, 0.87, 0.86, 0.86, 0.85, 0.85, 0.84, 0.84, 0.83, 0.83, 0.81, 0.81 and 0.80 respectively as those genes with the most powerful classifying potential to discriminate between TB cases and TST+ (**Fig 2A**). Genes that could best classify TB patients and TST- were IL7R, PRF1, NLRP1, CD3E, CCR7, FCGR1A, IL5, TLR9, BLR1, CD19, NLRP12, NOD2, PTPRCv1, GNLY, TLR5, NCAM1 and RAB33A with AUCs of 0.97, 0.94, 0.94, 0.93, 0.93, 0.93, 0.91, 0.89, 0.88, 0.87, 0.87, 0.83, 0.83, 0.81, 0.80, 0.80 and 0.80 respectively (**Fig 2B**). Transcriptomic profiles of those host genes (n = 15) that markedly classified active TB from both latent TB and healthy controls individuals (AUCs ≥ 0.80) are displayed in **Fig 3**. Genes that could discriminate TST+ from TST- were IL5, CCL22, TNF, IL23A and FCGR1A with AUCs of 0.84, 0.84, 0.82, 0.81 and 0.80 respectively (**Fig 2C**). Transcriptomic profiles of these genes that markedly classified latent TB and healthy controls are also displayed in **Fig 3**.

### Impact of anti-TB treatment (ATT) on the kinetic responses of the biomarkers associated with active TB

**Fig 4:**
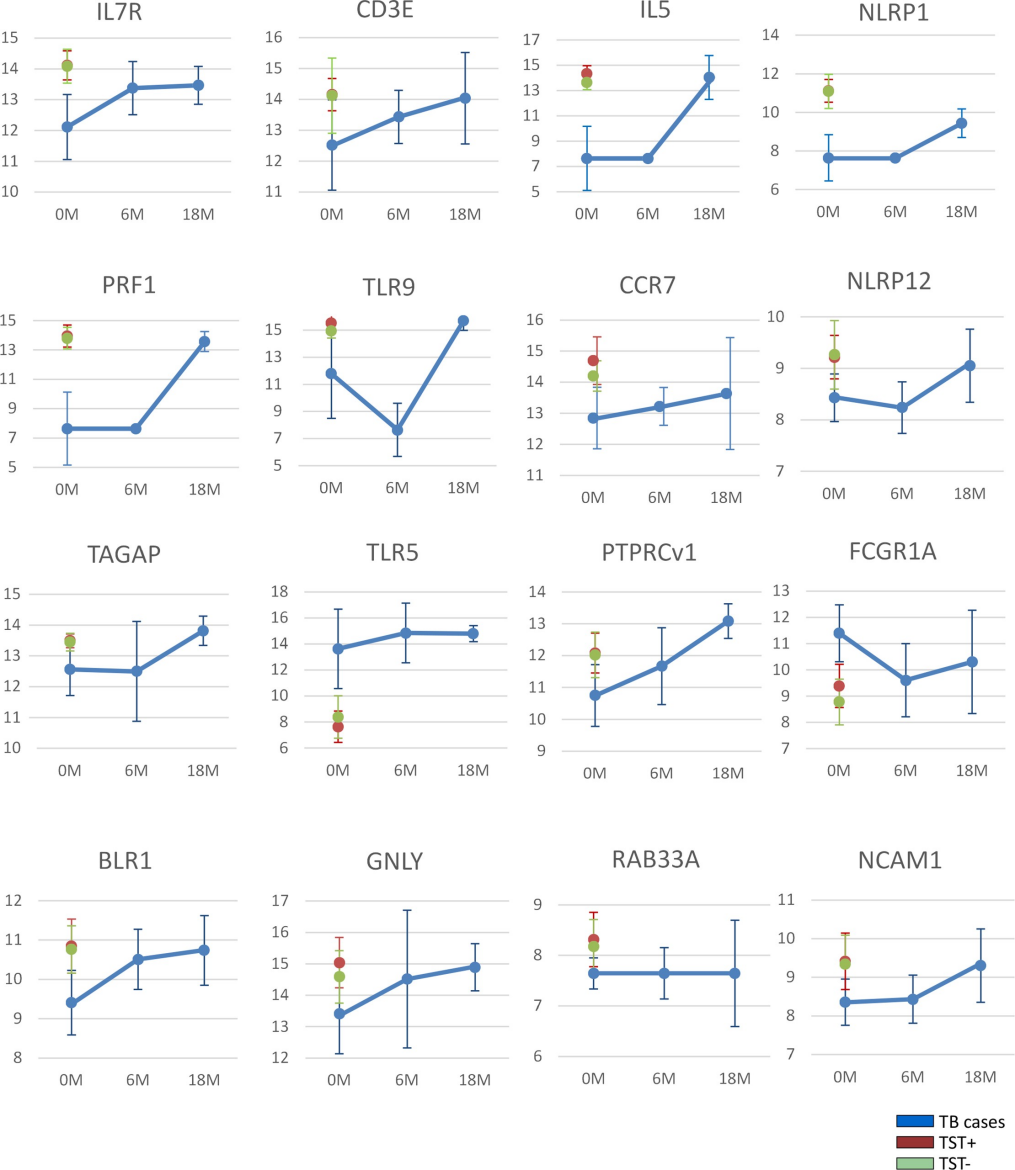
Kinetics of gene expression profiles in response to ATT treatment. Median gene expression levels (peak areas normalized to GAPDH and log2-transformed) and standard deviations are shown of the indicated genes at baseline (M0), 6 months (6M) and 18 months (18M) after anti-TB treatment (ATT) of HIV-negative subjects.

Next, we assessed the effect of ATT treatment on expression of the genes that markedly discriminated between TB cases versus TST+ and TST- controls at baseline. Thus, the gene expression of these markers in TB patients was measured at six months (6M) of ATT and compared to the baseline value (0M) of the same patients and with that of both control groups (TST+ and TST-). The expression levels of genes that markedly discriminated between TB cases versus TST+ and TST- at baseline partially normalized between baseline and 6M in TB patients following ATT treatment. Interestingly, the expression levels of many genes had not fully normalized to TST+ or TST- levels at the end of 6M of ATT therapy (**Fig 3 & Table 3**). Only the expression of 8 genes, including 4 transcripts which were among those with the most powerful potential to discriminate between TB disease and TST+ or TST- (PTPRCv1, FCGR1A, CASP8 and GNLY) (**Fig 2**), became indistinguishable from those of TST+ and TST- at the end of 6M ATT therapy (**Table 3**). However, most of the genes whose expression levels were not completely normalized yet at 6M did display expression levels that were indistinguishable from TST+ or TST- at 18 months follow up (**Table 4 & Fig 4**).

**Table 3:** Kinetic profiling of the response to TB treatment at 6M of ATT in active TB patients.

Gene Symbol	TB cases (0M)	TB cases (6M)	TST+ (0M)	TST- (0M)	TB cases (6M) vs TB cases (M0)	TB cases (6M) vs TST+ (M0)	TB cases (6M) vs TST- (M0)
**Immune cell subset markers**							
CD19	7.6(7.6–7.6)	9.7 (9.4–9.9)	7.7(7.6–8.1)	8.2(7.8–8.6)	**0.0000**	**0.0205**	0.7626
NCAM1	8.4(7.6–8.7)	8.4 (8.1–9.0)	9.4(8.8–9.9)	9.3(8.7–9.6)	0.5832	**0.0005**	**0.0045**
**T cell subset markers**							
CD3E	12.5(11.3–13.0)	13.4(13.0–14.1)	14.2(13.8–14.6)	14.1(13.7–14.3)	**0.0000**	**0.0015**	**0.0145**
CD4	12.0(11.4–12.5)	11.7(11.2–12.6)	12.8(12.4–13.3)	12.3(12.1–12.9)	0.3886	**0.0000**	**0.0068**
CD8A	12.7(12.3–13.0)	14.1(13.9–14.4)	13.0(12.8–13.4)	13.1(12.9–13.2)	**0.0000**	**0.0000**	**0.0000**
CCR7	12.8(12.2–13.4)	13.2(12.9–13.7)	14.7(14.1–15.0)	14.2(14.0–14.4)	0.0684	**0.0000**	**0.0000**
IL7R	12.1(11.7–13.0)	13.4 (12.6–13.8)	14.1(13.7–14.5)	14.1(13.8–14.4)	**0.0001**	**0.0000**	**0.0001**
PTPRCv1	10.7(10.2–11.3)	11.7 (11.1–12.1)	12.1(11.5–12.3)	12.0(11.3–12.2)	**0.0008**	0.0803	0.3470
AIRE	7.6(7.6–12.9)	12.8(12.4 to 13.3)	7.6(7.6–7.6)	7.6(7.6–7.6)	**0.0014**	**0.0000**	**0.0000**
**Th1/2/9/17 associated genes/Treg associated genes**							
IL2	9.1(8.4–9.6)	9.6(9.2–10.3)	10.0(9.4–10.7)	9.4(9.1–9.7)	**0.0135**	0.2009	0.3470
TNF	10.8(9.7–11.5)	12.3 (11.9–12.6)	10.2(10.0–10.5)	9.9(9.7–10.0)	**0.0000**	**0.0000**	**0.0001**
GATA3	7.6(7.6–7.6)	7.6 (7.6–7.7)	7.7(7.6–8.0)	7.8(7.6–8.3)	0.0841	**0.0448**	**0.0346**
IL5	7.6(7.6–12.1)	7.6 (7.6–7.6)	14.3(14.0–14.9)	13.6(13.4–13.8)	**0.0001**	**0.0000**	**0.0000**
IL13	7.6(7.6–7.6)	7.6 (7.6–7.6)	10.3(7.6–11.3)	8.9(7.6–10.3)	**0.0048**	**0.0000**	**0.0000**
CCL4	9.5(9.0–10.0)	10.4 (10.1–11.0)	10.1(10.0–10.3)	10.1(9.8–10.3)	**0.0000**	**0.0089**	**0.0016**
CTLA4	12.0(11.5–12.4)	11.8 (11.6–12.4)	12.6(12.2–12.7)	12.4(12.1–12.7)	0.4653	**0.0023**	**0.0049**
**Cytotoxicity genes**							
GNLY	13.4(12.9–14.2)	14.5 (13.9–15.2)	15.0(14.4–15.4)	14.6(14.3–15.0)	**0.0011**	0.0941	0.5225
GZMB	11.7(11.2–12.3)	12.2 (11.9–12.6)	12.6(12.0–13.0)	12.7(12.2–12.9)	**0.0068**	0.2886	0.1896
PRF1	7.6(7.6–12.3)	7.6 (7.6–7.6)	13.9(13.2–14.2)	13.8(13.2–14.2)	**0.0001**	**0.0000**	**0.0000**
**Apoptosis/survival**							
CASP8	12.3(11.9–12.7)	12.9 (12.5–13.2)	12.8(12.6–13.0)	12.9(12.5–13.1)	**0.0033**	0.9850	0.9850
BCL2	9.5(8.5–9.9)	10.1(9.6–10.4)	10.6(10.1–10.9)	10.2(10.0–11.0)	**0.0002**	**0.0060**	0.1185
TNFRSF1A	13.6(13.1–13.8)	13.5 (13.3–13.8)	13.9(13.7–14.2)	13.8(13.5–14.0)	0.7096	**0.0036**	**0.0462**
TNFRSF1B	11.4(10.8–11.8)	12.5 (12.1–12.8)	11.9(11.7–12.2)	11.5(11.3–11.9)	**0.0002**	**0.0048**	1.0000
**Myeloid associated genes**							
CD163	8.9(8.6–9.3)	7.6 (7.6–8.1)	9.3(9.0–9.6)	9.4(9.3–9.7)	**0.0000**	**0.0000**	**0.0000**
CCL2	7.9(7.6–9.4)	8.1 (7.6–9.2)	7.6(7.6–8.2)	7.6(7.6–7.6)	0.8752	**0.0324**	**0.0048**
CCL5	13.9(13.6–14.1)	14.0 (13.6–14.3)	14.6(14.3–15.0)	14.5(14.3–14.9)	0.7989	**0.0002**	**0.0019**
CCL22	13.9(12.3–15.3)	13.4 (12.2–14.2)	14.8(14.4–15.5)	13.9(13.8–14.2)	0.1959	**0.0000**	0.0575
CXCL13	10.8(10.2–11.2)	11.4 (10.9–11.9)	11.3(11.0–11.4)	11.1(10.7–11.3)	**0.0327**	0.3374	**0.0360**
IL12B	7.6(7.6–8.2)	7.6 (7.6–7.9)	8.6(8.3–9.1)	7.9(7.6–8.6)	0.8771	**0.0000**	**0.0248**
IL23A	11.4(11.3–11.7)	11.5 (11.4–11.7)	9.5(8.4–11.3)	11.3(11.3–11.4)	**0.0121**	**0.0000**	**0.0001**
**Pattern recognition receptors**							
MRC2	7.6(7.6–8.8)	9.6 (9.0–10.3)	7.6(7.6–7.6)	7.6(7.6–7.6)	**0.0000**	**0.0000**	**0.0000**
NOD2	8.9(8.0–9.5)	9.5 (9.1–9.8)	8.0(7.6–8.4)	7.7(7.6–8.0)	**0.0415**	**0.0000**	**0.0000**
TLR3	10.1(9.5–10.8)	10.0 (9.5–10.6)	9.4(9.3–9.8)	10.5(9.8–10.9)	0.9375	**0.0124**	0.1696
TLR5	13.6(8.8–15.1)	14.9 (14.1–15.3)	7.6(7.6–8.0)	8.4(7.6–10.7)	**0.0460**	**0.0000**	**0.0000**
TLR9	11.8(7.6–14.2)	7.6 (7.6–7.6)	15.5(15.1–15.9)	14.9(14.9–15.3)	**0.0007**	**0.0000**	**0.0001**
**Inflammasome components**							
NLRP1	7.6(7.6–9.5)	7.6 (7.6–7.6)	11.1(10.8–11.6)	11.1(10.7–11.5)	**0.0001**	**0.0000**	**0.0001**
NLRP2	11.1(10.0–12.0)	11.7 (11.4–12.2)	12.7(11.9–13.4)	11.7(11.6–12.1)	**0.0132**	**0.0007**	0.6248
NLRP12	8.4(8.0–8.7)	8.2 (7.9–8.5)	9.2(9.1–9.5)	9.3(8.9–9.5)	0.0900	**0.0000**	**0.0001**
NLRP13	7.6(7.6–8.9)	7.7 (7.6–8.4)	8.6(8.1–9.8)	8.1(7.6–8.9)	0.2677	**0.0006**	0.2327
**IFN signalling genes**							
FCGR1A	11.4(10.7–11.7)	9.6 (8.2–10.6)	9.4(9.1–10.4)	8.8(8.1–9.3)	**0.0004**	0.7777	0.0546
**Inflammation**							
TIMP2	14.3(13.5–14.7)	14.2 (13.9–14.5)	14.6(14.4–14.8)	14.5(14.2–14.7)	0.3779	**0.0020**	0.0803
**Other**							
AREG	7.6(7.6–12.1)	7.6 (7.6–7.6)	11.9(11.8–12.3)	12.0(11.5–12.4)	**0.0001**	**0.0000**	**0.0001**
TGFBR2	11.5(11.0–12.0)	12.2 (11.6–12.5)	11.9(11.7–12.3)	11.7(11.4–12.1)	**0.0002**	0.3666	0.0653
RAB13	8.2(7.6–8.8)	9.3 (8.9–9.9)	9.0(7.6–10.0)	9.4(8.4–9.8)	**0.0001**	0.4554	0.6718
RAB24	11.5(11.1–11.8)	11.3 (10.9–11.7)	11.2(10.8–11.5)	10.9(10.6–11.1)	0.1364	0.8656	**0.0187**
RAB33A	7.6(7.6–7.6)	7.6 (7.6–8.3)	8.3(7.8–8.8)	8.2(7.7–8.6)	0.0577	**0.0059**	**0.0320**
TAGAP	12.6(12.1–13.0)	12.5 (12.1–12.9)	13.5(13.3–13.5)	13.4(13.2–13.6)	0.1763	**0.0000**	**0.0001**
BPI	14.5(13.7–15.0)	13.6 (13.2–14.2)	15.2(14.9–15.4)	14.6(14.5–14.9)	**0.0311**	**0.0000**	**0.0001**
TWIST1	7.6(7.6–7.6)	7.6 (7.6–7.6)	7.6(7.6–8.0)	7.6(7.6–7.6)	0.9815	**0.0092**	0.5724
SEC14L1	13.9(13.7–14.3)	14.0 (13.5–14.5)	14.3(14.1–14.9)	14.2(13.9–14.8)	0.9844	**0.0252**	**0.0385**
BLR1	9.4(9.2–10.1)	10.5 (10.1–11.0)	10.8(10.3–11.1)	10.8(10.5–11.4)	**0.0002**	0.2076	**0.0368**

Median (inter quartile range) gene expression values (peak areas normalized for GAPDH and log2-transformed) are shown. Significant differences between active TB patients at baseline (0M) and 6 months following ATT treatment initiation (6M) were determined using Wilcoxon signed-rank test. Significant differences between active TB at the 6M and TST+ or TST- at the 0M time point was determined using Wilcoxon Mann-Whitney test. In red, genes are indicated that were more highly expressed in the test group compared to the reference/control group whereas in blue genes are indicated that had lower expression in the test group compared to the reference/control group. Genes listed in this table were differentially expressed between any of the study groups at baseline (0M) (Table 2). P-values ≤ 0.05 are indicated in bold.

**Table 4:** Kinetic profiling of the response to TB treatment after completed ATT in active TB patients (18M).

Gene Symbol	TB cases (0M)	TB cases (18M)	TST+ (0M)	TST- (0M)	TB cases (18M) vs TB cases (0M)	TB cases (18M) vs TST+ (0M)	TB cases (18M) vs TST- (0M)
**Immune cell subset markers**							
CD19	7.6(7.6–7.6)	8.7 (8.3–9.0)	7.7(7.6–8.1)	8.2(7.8–8.6)	**0.0001**	**0.0022**	0.0581
NCAM1	8.4(7.6–8.7)	9.3(8.7–10.0)	9.4(8.8–9.9)	9.3(8.7–9.6)	**0.0009**	0.9709	0.6968
**T cell subset markers**							
CD3E	12.5(11.3–13.0)	14.0(13.7–14.5)	14.2(13.8–14.6)	14.1(13.7–14.3)	**0.0000**	0.4203	0.9198
CD4	12.0(11.4–12.5)	12.8(12.6–13.2)	12.8(12.4–13.3)	12.3(12.1–12.9)	**0.0042**	0.6091	**0.0302**
CD8A	12.7(12.3–13.0)	14.0(13.7–14.3)	13.0(12.8–13.4)	13.1(12.9–13.2)	**0.0000**	**0.0000**	**0.0000**
CCR7	12.8(12.2–13.4)	13.6(13.2–14.0)	14.7(14.1–15.0)	14.2(14.0–14.4)	**0.0012**	**0.0032**	**0.0052**
IL7R	12.1(11.7–13.0)	13.7(13.2–14.2)	14.1(13.7–14.5)	14.1(13.8–14.4)	**0.0000**	**0.0051**	**0.0130**
PTPRCv1	10.7(10.2–11.3)	13.1(12.5–13.5)	12.1(11.5–12.3)	12.0(11.3–12.2)	**0.0000**	**0.0000**	**0.0000**
AIRE	7.6(7.6–12.9)	8.3(7.64–9.1)	7.6(7.6–7.6)	7.6(7.6–7.6)	**0.0388**	**0.0000**	**0.0000**
**Th1/2/9/17 associated genes/Treg associated genes**							
IL2	9.1(8.4–9.6)	10.3(9.5–11.0)	10.0(9.4–10.7)	9.4(9.1–9.7)	**0.0009**	0.5921	**0.0235**
TNF	10.8(9.7–11.5)	10.0(12.3–14.4)	10.2(10.0–10.5)	9.9(9.7–10.0)	0.6926	**0.0027**	**0.0000**
GATA3	7.6(7.6–7.6)	8.1(7.64–8.3)	7.7(7.6–8.0)	7.8(7.6–8.3)	**0.0001**	0.0873	0.2960
IL5	7.6(7.6–12.1)	14.0(12.3–14.4)	14.3(14.0–14.9)	13.6(13.4–13.8)	**0.0000**	**0.0251**	0.3181
IL13	7.6(7.6–7.6)	7.64(7.64–9.0)	10.3(7.6–11.3)	8.9(7.6–10.3)	0.2937	**0.0118**	0.0932
CCL4	9.5(9.0–10.0)	10.7(10.3–11.3)	10.1(10.0–10.3)	10.1(9.8–10.3)	**0.0001**	**0.0020**	**0.0003**
CTLA4	12.0(11.5–12.4)	13.3(13.1–13.6)	12.6(12.2–12.7)	12.4(12.1–12.7)	**0.0000**	**0.0000**	**0.0000**
**Cytotoxicity genes**							
GNLY	13.4(12.9–14.2)	14.9(14.0–11.3)	15.0(14.4–15.4)	14.6(14.3–15.0)	**0.0003**	0.6091	0.3301
GZMB	11.7(11.2–12.3)	12.5(12.1–13.0)	12.6(12.0–13.0)	12.7(12.2–12.9)	**0.0029**	0.7332	0.6789
PRF1	7.6(7.6–12.3)	13.6(10.3–11.3)	13.9(13.2–14.2)	13.8(13.2–14.2)	**0.0000**	0.3676	0.5590
**Apoptosis/survival**							
CASP8	12.3(11.9–12.7)	13.3(13.0–13.5)	12.8(12.6–13.0)	12.9(12.5–13.1)	**0.0000**	**0.0038**	**0.0030**
BCL2	9.5(8.5–9.9)	10.4(9.9–10.7)	10.6(10.1–10.9)	10.2(10.0–11.0)	**0.0001**	0.3941	0.8171
TNFRSF1A	13.6(13.1–13.8)	14.1(13.9–14.3)	13.9(13.7–14.2)	13.8(13.5–14.0)	**0.0011**	0.2172	**0.0136**
TNFRSF1B	11.4(10.8–11.8)	12.8(12.6–13.2)	11.9(11.7–12.2)	11.5(11.3–11.9)	**0.0002**	**0.0000**	**0.0000**
**Myeloid associated genes**							
CD163	8.9(8.6–9.3)	8.1(7.8–8.4)	9.3(9.0–9.6)	9.4(9.3–9.7)	**0.0262**	**0.0000**	**0.0000**
CCL2	7.9(7.6–9.4)	9.5(8.8–10.0)	7.6(7.6–8.2)	7.6(7.6–7.6)	**0.0010**	**0.0000**	**0.0000**
CCL5	13.9(13.6–14.1)	14.9(14.3–15.1)	14.6(14.3–15.0)	14.5(14.3–14.9)	**0.0000**	0.4504	0.1375
CCL22	13.9(12.3–15.3)	14.8(13.9–15.2)	14.8(14.4–15.5)	13.9(13.8–14.2)	0.1808	0.3181	**0.0221**
CXCL13	10.8(10.2–11.2)	12.8(12.5–13.3)	11.3(11.0–11.4)	11.1(10.7–11.3)	**0.0000**	**0.0000**	**0.0000**
IL12B	7.6(7.6–8.2)	7.64(7.64–7.9)	8.6(8.3–9.1)	7.9(7.6–8.6)	0.9373	**0.0007**	**0.0793**
IL23A	11.4(11.3–11.7)	11.5(9.9–11.6)	9.5(8.4–11.3)	11.3(11.3–11.4)	0.9273	**0.0013**	0.2054
**Pattern recognition receptors**							
MRC2	7.6(7.6–8.8)	8.6(8.2–10.1)	7.6(7.6–7.6)	7.6(7.6–7.6)	**0.0006**	**0.0000**	**0.0000**
NOD2	8.9(8.0–9.5)	9.4(9.2–10.2)	8.0(7.6–8.4)	7.7(7.6–8.0)	**0.0074**	**0.0000**	**0.0000**
TLR3	10.1(9.5–10.8)	9.2(8.5–9.6)	9.4(9.3–9.8)	10.5(9.8–10.9)	**0.0014**	**0.0194**	**0.0004**
TLR5	13.6(8.8–15.1)	14.8(14.3–15.3)	7.6(7.6–8.0)	8.4(7.6–10.7)	0.1070	**0.0000**	**0.0000**
TLR9	11.8(7.6–14.2)	15.7(14.9–16.1)	15.5(15.1–15.9)	14.9(14.9–15.3)	**0.0000**	0.6436	0.0575
**Inflammasome components**							
NLRP1	7.6(7.6–9.5)	9.4(8.7–9.7)	11.1(10.8–11.6)	11.1(10.7–11.5)	0.0703	**0.0000**	**0.0000**
NLRP2	11.1(10.0–12.0)	12.9(12.6–13.2)	12.7(11.9–13.4)	11.7(11.6–12.1)	**0.0000**	0.3423	**0.0000**
NLRP12	8.4(8.0–8.7)	9.1(8.4–9.3)	9.2(9.1–9.5)	9.3(8.9–9.5)	**0.0065**	0.0543	0.1648
NLRP13	7.6(7.6–8.9)	8.7(7.64–9.5)	8.6(8.1–9.8)	8.1(7.6–8.9)	0.8900	0.6429	0.2560
**IFN signalling genes**							
FCGR1A	11.4(10.7–11.7)	10.3(7.64–11.1)	9.4(9.1–10.4)	8.8(8.1–9.3)	**0.0051**	0.4494	0.0504
**Inflammation**							
TIMP2	14.3(13.5–14.7)	14.6(14.4–14.9)	14.6(14.4–14.8)	14.5(14.2–14.7)	0.1361	0.8076	0.0883
**Other**							
AREG	7.6(7.6–12.1)	10.8(10.7–11.3)	11.9(11.8–12.3)	12.0(11.5–12.4)	0.6157	**0.0000**	**0.0001**
TGFBR2	11.5(11.0–12.0)	12.9(12.1–13.1)	11.9(11.7–12.3)	11.7(11.4–12.1)	**0.0000**	**0.0011**	**0.0001**
RAB13	8.2(7.6–8.8)	9.9(9.7–10.3)	9.0(7.6–10.0)	9.4(8.4–9.8)	**0.0000**	**0.0029**	**0.0010**
RAB24	11.5(11.1–11.8)	11.6(11.3–12.0)	11.2(10.8–11.5)	10.9(10.6–11.1)	0.1997	**0.0118**	**0.0000**
RAB33A	7.6(7.6–7.6)	7.64(7.64–7.74)	8.3(7.8–8.8)	8.2(7.7–8.6)	0.3569	**0.0015**	**0.0034**
TAGAP	12.6(12.1–13.0)	13.8(13.5–14.3)	13.5(13.3–13.5)	13.4(13.2–13.6)	**0.0001**	**0.0007**	**0.0007**
BPI	14.5(13.7–15.0)	14.8(13.7–15.1)	15.2(14.9–15.4)	14.6(14.5–14.9)	0.1808	**0.0041**	0.8838
TWIST1	7.6(7.6–7.6)	7.6(7.6–7.6)	7.6(7.6–8.0)	7.6(7.6–7.6)	0.3173	**0.0053**	1.0000
SEC14L1	13.9(13.7–14.3)	14.5(14.2–15.0)	14.3(14.1–14.9)	14.2(13.9–14.8)	**0.0009**	0.4217	0.1375
BLR1	9.4(9.2–10.1)	10.7(10.5–11.1)	10.8(10.3–11.1)	10.8(10.5–11.4)	**0.0007**	0.9612	0.8265

Median (inter quartile range) gene expression values (peak areas normalized for GAPDH and log2-transformed) are shown. Significant differences between active TB patients at 18 months following ATT treatment initiation (18M) and baseline (0M) were determined using Wilcoxon signed-rank test. Significant differences between active TB at the 18M and TST+ or TST- at the 0M time point was determined using Wilcoxon Mann-Whitney test. In red genes are indicated that were more highly expressed in the test group compared to the reference/control group whereas in blue genes are indicated that had lower expression in the test group compared to the reference/control group. Genes listed in this table were differentially expressed between any of the study groups at baseline (0M) (Table 2). P-values ≤ 0.05 are indicated in bold.

### Different gene networks discriminate TB cases from controls in HIV-positive and HIV-negative individuals

Out of the 48 genes which were significantly differentially expressed between TB cases and TST+ subjects in this HIV-negative cohort, only 7 genes (CD4, PTPRCv1, TLR3, TNFRSF1A, NLRP12, BLR1 and FCGR1A) were significantly different between HIV-positive TB cases and TST+ individuals in our previous study in the same location [24]. Moreover, the expression of TNFRSF1A, TLR3 and NLRP12 was significantly higher in TB cases than TST+ controls during HIV coinfection, in contrast to the results obtained here in HIV negative individuals. Similarly, only 12 out of the 39 host genes which were significantly differentially expressed between TB cases and TST- in HIV negative individuals, including FCGR1A, RAB24, CD3E, CD4, IL7R, PTPRCv1, GNLY, GZMB, TNFRSF1A, CCL5, NLRP12 and BLR1, were also significantly different between TB cases and TST- in HIV coinfected individuals in our previous study [24], and again the expression of TNFRSF1A and NLRP12 was significantly higher in TB cases than TST- controls during HIV coinfection, in contrast to the results obtained here in HIV negative individuals. None of the 17 host genes which were significantly differentially expressed between HIV-negative TST+ and TST- individuals was significantly different in HIV positive TST+ and TST- individuals in our previous study [24].

**Fig 5:**
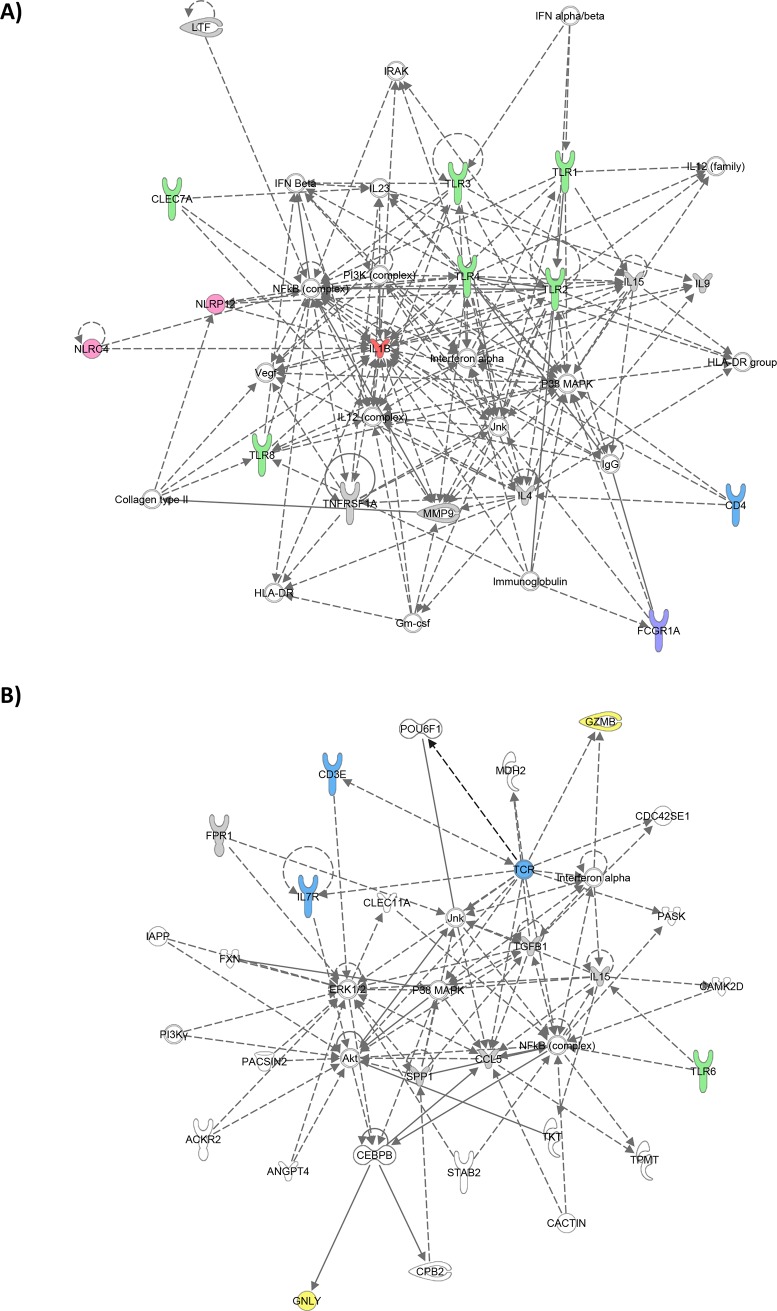
IPA network analysis in HIV-positive TB cohort. Ingenuity Pathway Analysis in HIV-positive individuals of (A) TB-associated genes that were differentially expressed between TB cases and TST+ individuals as well as between TB cases and TST- individuals at baseline and (B) genes that were differentially expressed between TST+ and TST- individuals at baseline. Dark blue: T cell associated genes, Yellow: Cytotoxicity associated genes, Green: Pattern recognition receptors, Purple: IFN-inducible genes, Pink: Inflammasome components, Red: pro-inflammatory cytokines.

Ingenuity Pathway Analysis of the data from the HIV-positive cohort in the previous study [24] revealed an over-representation of pattern recognition receptors including TLR2 and TLR4 (**Fig 5A**) in TB-associated genes which was not seen in the HIV-negative cohort (**Fig 1A**). The comparison of HIV-positive TST+ and TST- individuals revealed a central role for cytotoxicity and T cell genes (**Fig 5B**) in contrast to the dominance of pro-inflmmatory cytokines seen in HIV-negative individuals (**Fig 1B**).

## Discussion

Assessing the consistency of previously identified candidate biomarkers and finding additional candidate genes for diagnosing TB disease and for monitoring treatment responses will be important for the future direction of TB disease control. Here, we identified gene expression patterns which could discriminate clinical stages of TB, using a focused gene expression profiling platform, dcRT-MLPA [18], targeting innate and adaptive immune response genes, to analyze RNA expression levels of 105 pre-selected genes in peripheral blood. The gene expression of 15 genes with AUCs ≥0.80 (IL7R, CD3E, IL5, NLRP1, PRF1, TLR9, CCR7, NLRP12, TLR5, PTPRCv1, FCGR1A, BLR1, GNLY, RAB33A and NCAM1) was strongly associated with TB disease and these genes indeed play critical roles in the immune response against TB. There was a clear association between TB disease and low BMI in this cohort: observed gene expression differences might be related to nutritional status but this is intrinsically linked to disease profile in TB.

Expression of TLR9, NLRP1, NLPR12, RAB33A and BLR1 was significantly lower in TB patients compared to TST+ and TST- subjects, in agreement with published data [18,25,26,27]. Toll-like receptors (TLR) play a critical role in the innate immune response to exogenous pathogens. Low expression of TLR9 has a critical role in TB incidence and progression, and this might be associated with combined defects in pro-inflammatory cytokine production such as IFN-γ recall responses [26]. Low expression of NLRP1 and NLRP12 might be related to a risk of susceptibility for bacterial diseases, via reduced cleavage of pro-IL-1β and pro-IL-18 to produce mature isoforms [28], and via avoidance of infected macrophage lysis [29] which contributes to pathology in TB. Rab33A is a novel CD8^+^ T cell factor and the expression may involved in susceptibility to TB disease [27].

The observed lower expression of T cell associated genes (e.g. IL7R, CD3E, CCR7 and PTPRCv1) in TB patients has been shown previously [21,30] and might be associated with reactivation of infection and migration of cells to the site of infection [31]. Similarly, lower expression of other immune subset genes (such as NK marker NCAM1) in blood in TB patients may also relate to migration of lymphocytes or natural killer cells from the peripheral blood to the site of infection [32]. Furthermore, GNLY and PRF1 expression levels were also significantly lower in TB patients compared to TST+ and TST- individuals, which is consistent with published data [33,34] and might be explained by rapid consumption of both perforin and granulysin during active disease due to an ongoing effector immune response, or due to migration of the T cell subset responsible for its production [35].

FCGR1A and TLR5 were also found to be differentially expressed between TB cases and TST+ or TST- individuals, in agreement with published data [36,37,38,39]. However, these genes were higher expressed in TB patients compared to controls and were found to constitute the best discriminatory power between TB cases versus both TST+ and TST- controls. FCGR1A is an essential component of interferon signalling and plays a central role in endocytosis, phagocytosis, antibody-dependent cellular toxicity, cytokine release, and superoxide generation [40] but may also participate in TB pathogenesis. In contrast, TLR5 is expressed in myeloid cells during TB infection and its role may associate with an imbalance in Th1 and Th2 cells by increasing the expression of IL-4 [41].

We also assessed the expression levels of host genes in response to ATT. We showed that expression levels of a subset of genes that markedly discriminated between TB cases versus TST+ and/or TST- controls at baseline were normalized in ATT treated TB patients at 6 months. However, in contrast to most previous studies in which normalization was completed between 2 and 6 months of treatment [42,43], the majority of the genes in our study were only fully normalized at the 18 months follow-up time point. Treatment-response transcriptomic signatures can significantly change already within 1 week of treatment [44], and continue to change until the end of ATT treatment at 6 months [18,45] and even after treatment is completed [11,46]. The expression of only a small number of genes, including PTPRCv1, FCGR1A, GZMB, CASP8 and GNLY, fully returned to the expression levels observed in TST+ and TST- subjects after the full 6 months of treatment in this study. Differential expression of gene profiles in TB patients during 6 months anti-TB chemotherapy compared to baseline has previously been reported [42,43,47] and correlated with a clearance of actively dividing bacilli load [44]. However, TB cases with clinically curative treatment at the end of 6 months therapy may not have completely cleared the infection yet, and may not have reached the end of the disease pathology resolution process due to the presence of few remaining viable *Mtb*, with the potential to elicit a host response [48] as well as ongoing immunopathology in sterilized lesions.

There were some notable differences in discriminating TB cases from controls using the expression of immune-related genes amongst HIV-positive [24] and -negative individuals (this study). The discriminatory potential of genes identified in HIV-negative individuals using ROC included immune cell markers (NCAM1), T cell associated genes (IL7R, CD3E, CCR7, PTPRCv1), T helper type 2 associated genes (IL5), cytotoxicity genes (GNLY and PRF1), pattern recognition receptors (TLR5 and TLR9), inflamasome components (NLRP1 and NLRP12), IFN signalling genes (FCGR1A), GTPase activating genes (RAB33A) and G-protein couple receptors (BLR1) (Fig 2A and 2B). With the exception of FCGR1A, all of these genes did not have discriminatory potential amongst HIV-positive individuals using ROC cutoff ≥ 0.80 [24]. Pattern recognition receptors, including TLR2 and TLR4, were over-represented in network analysis of TB-associated genes in HIV-positive individuals (Fig 5A) which was not the case in HIV-negative individuals (Fig 1A), revealing fundamental differences in biological response and biomarker expression in these cohorts. In previous studies, TB patients without HIV infection showed no difference in TLR2 and TLR4 expression in monocytes compared to healthy donors [49] but TLR2 and TLR4 are most strongly up-regulated in mDCs of TB patients coinfected with HIV [50] consistent with the findings in this report. Using ROC cutoff ≥ 0.80, the expression of FCGR1A was the only marker consistently identfied in both HIV-positive and -negative individuals which is consistent with a previous report by Sutherland et al [30]. The dominance of pro-inflammatory cytokines seen in HIV-negative LTBI may be related to activation of T cells [51] which may contribute to containment of *Mtb* infection. In contrast, low expression of cytotoxicity genes and T cell-associated genes observed in HIV-positive LTBI may reflect enhanced recruitment of T cells to the site of *Mtb* infection[52], or deletion of the activated T cells [53], which may contribute to HIV disease progression and exacerbate the HIV epidemic.

There were also notable differences between this report and a previous report in the context of Ethiopia. While only 9 of 45 host genes genes measured by Mihret *et al*. had significantly different expression between active TB cases and household contacts [21], 21 out of these 45 host genes had significantly differencial expression in TB cases compared to both TST+ and TST- subjects in our study. The expression of FCGR1A and IL7R were the only TB-associated markers that were consistently differentially expressed between TB patients and control groups in our study compared to the previous study in the context of Ethiopia and this may be attributable to the selection criteria for the control groups [30] which consisted of household contacts in Mihret *et al*. and daily laborers in our study, or may reflects huge genetic heterogeneity amongst the Ethiopian population. Moreover, 5 out of 45 host genes measured by Mihret *et al*. [21] showed differential expression between latent TB infected and uninfected individuals, whereas 7 of the 45 host genes was differentially expressed between latent TB infected and uninfected individuals in our study. However, there was no overlap in the genes discriminating between TST+ and TST- individuals in both studies.

In conclusion, the expression levels of 15 host genes (IL7R, CD3E, IL5, NLRP1, PRF1, TLR9, CCR7, NLRP12, TLR5, PTPRCv1, FCGR1A, BLR1, GNLY, RAB33A and NCAM1) in peripheral blood can discriminate active TB disease from latent TB infection and uninfected controls in an HIV-negative cohort. However, almost all these markers, except for FCGR1A, can not discriminate between active and latent TB in TB-HIV co-infected subjects. Our data also show that complex gene expression signatures are required to fully measure changes in blood transcriptomes during and after successful ATT, such that a combination including those which resolve completely during the 6-months treatment phase of therapy (PTPRCv1, FCGR1A, GZMB, CASP8 and GNLY) and those which only fully return to normal levels during the post-treatment resolution phase, might be required to fully characterise drug-induced relapse-free cure. Further research is needed to completely charaterise the optimal complex signature in different populations and larger study populations.

## Supporting information

S1 Table List of target genes for dcRT-MLPA. 105 selected genes and 4 housekeeping genes to profile innate and adaptive immune responses.
(DOC)
