# Supplementary material for: Gene expression profiles classifying clinical stages of tuberculosis and monitoring treatment responses in Ethiopian HIV-negative and HIV-positive cohorts
Source: PLoS One. 2019 Dec 10;14(12):e0226137. doi: 10.1371/journal.pone.0226137 (PMC6903757; doi:10.1371/journal.pone.0226137)
Supplement: S1 Table — 105 selected genes and 4 housekeeping genes to profile innate and adaptive immune responses. (DOC) [file pone.0226137.s001.doc]

| **Table S1. List of target genes for dcRT-MLPA.** | | | | |  |  |
| --- | --- | --- | --- | --- | --- | --- |
| **Immune cell subset markers** |  | **Treg associated genes** |  | **Chemokines** |  | **Inflammation** |
| CD19 ^1^ |  | CCL4^1, 2^ |  | CCL13 ^3^ |  | MMP9 ^1, 4^ |
| NCAM1^5^ |  | CTLA4 ^6^ |  | CCL19 ^7, 8^ |  | SPP1 ^9^ |
| **T cell subset markers** |  | FOXP3^1, 10^ |  | **Pattern recognition receptors** |  | TIMP2^1, 4^ |
| CD3E ^1, 4, 11^ |  | IL2RA ^1, 12^ |  | CD209 ^13^ |  | **IFN signaling genes** |
| CD4 ^1, 11^ |  | LAG3 ^14^ |  | CLEC7A ^13^ |  | FCGR1A ^4, 7, 9^ |
| CD8A^1, 4, 11^ |  | TGFB1^1, 2^ |  | MRC1 ^15^ |  | **Cell Growth/proliferation** |
| CCR7 ^1, 11^ |  | TNFRSF18 ^14^ |  | MRC2 ^15^ |  | AREG ^16^ |
| IL7R ^1, 4, 7^ |  | **Cytotoxicity markers** |  | NOD1^15^ |  | TGFBR2 ^1, 4^ |
| PTPRCv1 ^17, 18^ |  | GNLY ^9^ |  | NOD2 ^9, 13^ |  | **Small GTPases/(Rho) GTPaseactivating proteins** |
| PTPRCv2 ^17^ |  | GZMA ^9^ |  | TLR1 ^13, 19^ |  | RAB13 ^1^ |
| AIRE ^20^ |  | GZMB ^9^ |  | TLR2 ^9, 13, 19, 21^ |  | RAB24 ^1, 4^ |
| **Th1 associated genes** |  | PRF1 ^9^ |  | TLR3 ^13^ |  | RAB33A ^1^ |
| CXCL10 ^1, 11, 12^ |  | **Apoptosis/survival** |  | TLR4 ^2, 9, 19, 21^ |  | TAGAP ^18, 22^ |
| IFNG ^10-12^ |  | CASP8 ^1, 4^ |  | TLR5 ^9, 13^ |  | TBC1D7 ^23, 24^ |
| IL1B ^9, 10, 19^ |  | BCL2 ^1, 7, 9^ |  | TLR6 ^9^ |  | **Anti-microbial activity** |
| IL2 ^10, 12^ |  | FASLG ^1, 9^ |  | TLR7 ^11^ |  | BPI ^2, 4, 9^ |
| IL15 ^20^ |  | FLCN1 ^20^ |  | TLR8 ^9, 21^ |  | LTF ^1, 7, 9^ |
| TBX21 ^2^ |  | TNFRSF1A^1^ |  | TLR9 ^2, 19, 21^ |  | **E3 ubiquitine protein ligase** |
| TNF ^1^ |  | TNFRSF1B ^1, 4^ |  | TLR10 ^25^ |  | NEDD4L^20^ |
| **Th2 associated genes** |  | **Myeloid associated genes** |  | **Inflammasome components** |  | **Scavenger receptor** |
| GATA3 ^18, 26^ |  | CD14 ^1, 4^ |  | NLRC4 ^2, 27^ |  | MARCO^7, 9^ |
| IL4 ^1, 4^ |  | CD163 ^18, 28, 29^ |  | NLRP1^27, 30^ |  | **Transcriptional regulators/activators** |
| IL4δ2 ^7, 31^ |  | CCL2 ^11, 15^ |  | NLRP2 ^32^ |  | CAMTA1^33, 34^ |
| IL5 ^35^ |  | CCL5 ^15^ |  | NLRP3^27, 30^ |  | TWIST1 ^36^ |
| IL6 ^10^ |  | CCL22 ^14^ |  | NLRP4 ^30, 37^ |  | ZNF331 ^2^ |
| IL10 ^35, 38^ |  | CXCL13 ^8^ |  | NLRP6 ^32, 39^ |  | ZNF532 ^40^ |
| IL13 ^2^ |  | IL12A ^2, 10^ |  | NLRP7 ^32^ |  | **Intracellular transport** |
| **Th9 associated genes** |  | IL12B ^10^ |  | NLRP10 ^41, 42^ |  | SEC14L1^4^ |
| IL9 |  | IL23A ^2^ |  | NLRP11 ^43^ |  | **G-protein-couples receptors** |
| **Th17 associated genes** |  | FPR1 ^1, 4^ |  | NLRP12 ^44-47^ |  | BLR1^1, 4, 7^ |
| IL17A ^48, 49^ |  |  |  | NLRP13^50^ |  | **Reference genes** |
| IL22RA1^12^ |  |  |  |  |  | ABR |
| RORC ^48^ |  |  |  |  |  | B2M |
|  |  |  |  |  |  | GAPDH |
|  |  |  |  |  |  | GUSB |

**Reference**

**1.** Joosten SA, Goeman JJ, Sutherland JS, et al. Identification of biomarkers for tuberculosis disease using a novel dual-color RT-MLPA assay. *Genes Immun.*13(1):71-82.

**2.** Tientcheu LD, Haks MlC, Agbla SC, et al. Host Immune Responses Differ between M. africanum- and M. tuberculosis-Infected Patients following Standard Anti-tuberculosis Treatment. *PLOS Neglected Tropical Diseases.*10(5):e0004701.

**3.** Dutta NK, Mehra S, Martinez AN, et al. The Stress-Response Factor SigH Modulates the Interaction between Mycobacterium tuberculosis and Host Phagocytes. *PLoS ONE.*7(1):e28958.

**4.** Jenum S, Dhanasekaran S, Lodha R, et al. Approaching a diagnostic point-of-care test for pediatric tuberculosis through evaluation of immune biomarkers across the clinical disease spectrum. *Scientific reports.*6:18520-18520.

**5.** Jacobs R, Malherbe S, Loxton AG, et al. Identification of novel host biomarkers in plasma as candidates for the immunodiagnosis of tuberculosis disease and monitoring of tuberculosis treatment response. *Oncotarget.*7(36):57581-57592.

**6.** Wang C, Jiang T, Wei L, et al. Association of CTLA4 gene polymorphisms with susceptibility and pathology correlation to pulmonary tuberculosis in Southern Han Chinese. *International journal of biological sciences.*8(7):945-952.

**7.** Mihret A, Loxton AG, Bekele Y, et al. Combination of gene expression patterns in whole blood discriminate between tuberculosis infection states. *BMC Infectious Diseases.* 2014;14(1):257.

**8.** Slight SR, Khader SA. Chemokines shape the immune responses to tuberculosis. *Cytokine & growth factor reviews.*24(2):105-113.

**9.** Maertzdorf J, Repsilber D, Parida SK, et al. Human gene expression profiles of susceptibility and resistance in tuberculosis. *Genes Immun.* 2011;12.

**10.** Wu B, Huang C, Kato-Maeda M, et al. Messenger RNA Expression of IL-8, FOXP3, and IL-12β Differentiates Latent Tuberculosis Infection from Disease. *Journal of immunology (Baltimore, Md. : 1950).* 2007;178(6):3688-3694.

**11.** Berry MP, Graham CM, McNab FW, et al. An interferon-inducible neutrophil-driven blood transcriptional signature in human tuberculosis. *Nature.* Aug 19 2010;466(7309):973-977.

**12.** Lu C, Wu J, Wang H, et al. Novel biomarkers distinguishing active tuberculosis from latent infection identified by gene expression profile of peripheral blood mononuclear cells. *PLoS ONE.* 2011;6.

**13.** Kleinnijenhuis J, Oosting M, Joosten LAB, Netea MG, Van Crevel R. Innate Immune Recognition of Mycobacterium tuberculosis. *Clinical and Developmental Immunology.*2011:12.

**14.** Lienhardt C, Azzurri A, Amedei A, et al. Active tuberculosis in Africa is associated with reduced Th1 and increased Th2 activity in vivo. *European Journal of Immunology.* 2002;32(6):1605-1613.

**15.** Azad AK, Sadee W, Schlesinger LS. Innate immune gene polymorphisms in tuberculosis. *Infection and immunity.*80(10):3343-3359.

**16.** Hemingway C, Berk M, Anderson ST, et al. Childhood tuberculosis is associated with decreased abundance of T cell gene transcripts and impaired T cell function. *PLoS ONE.*12(11):e0185973.

**17.** Montes J, Gambon-Deza F, Pacheco M, Cerda T. [Memory T lymphocytes during infection and tuberculosis infection and disease]. *Arch Bronconeumol.* Sep 1998;34(8):384-387.

**18.** Geluk A, van Meijgaarden KE, Wilson L, et al. Longitudinal immune responses and gene expression profiles in type 1 leprosy reactions. *J Clin Immunol.* Feb 2014;34(2):245-255.

**19.** Mortaz E, Adcock IM, Tabarsi P, et al. Interaction of Pattern Recognition Receptors with Mycobacterium Tuberculosis. *Journal of clinical immunology.*35(1):1-10.

**20.** Sloot R, Schim van der Loeff MF, van Zwet EW, et al. Biomarkers Can Identify Pulmonary Tuberculosis in HIV-infected Drug Users Months Prior to Clinical Diagnosis. *EBioMedicine.*2(2):172-179.

**21.** Faridgohar M, Nikoueinejad H. New findings of Toll-like receptors involved in Mycobacterium tuberculosis infection. *Pathogens and global health.*111(5):256-264.

**22.** Arshad M, Bhatti A, John P. Identification and in silico analysis of functional SNPs of human TAGAP protein: A comprehensive study. *PLoS ONE.*13(1):e0188143.

**23.** Dibble CC, Elis W, Menon S, et al. TBC1D7 is a third subunit of the TSC1-TSC2 complex upstream of mTORC1. *Mol Cell.* Aug 24;47(4):535-546.

**24.** Pan H, Zhong X-p, Lee S. Sustained activation of mTORC1 in macrophages increases AMPKÎ±-dependent autophagy to maintain cellular homeostasis. *BMC Biochemistry.*17(1):14.

**25.** Wang Y, Zhang MM, Huang WW, et al. Polymorphisms in Toll-Like Receptor 10 and Tuberculosis Susceptibility: Evidence from Three Independent Series. *Front Immunol.*9:309.

**26.** da Silva MV, Massaro Junior VJ, Machado JR, et al. Expression pattern of transcription factors and intracellular cytokines reveals that clinically cured tuberculosis is accompanied by an increase in Mycobacterium-specific Th1, Th2, and Th17 cells. *BioMed research international.*2015:591237-591237.

**27.** Tan HY, Yong YK, Shankar EM, et al. Aberrant Inflammasome Activation Characterizes Tuberculosis-Associated Immune Reconstitution Inflammatory Syndrome. *The Journal of Immunology.*196(10):4052-4063.

**28.** Suzuki Y, Shirai M, Asada K, et al. Utility of Macrophage-activated Marker CD163 for Diagnosis and Prognosis in Pulmonary Tuberculosis. *Annals of the American Thoracic Society.*14(1):57-64.

**29.** Olson A, Ragan EJ, Nakiyingi L, et al. Brief Report: Pulmonary Tuberculosis Is Associated With Persistent Systemic Inflammation and Decreased HIV-1 Reservoir Markers in Coinfected Ugandans. *Journal of acquired immune deficiency syndromes (1999).*79(3):407-411.

**30.** PÃ©trilli V, Dostert C, Muruve DA, Tschopp Jr. The inflammasome: a danger sensing complex triggering innate immunity. *Current Opinion in Immunology.* 2007;19(6):615-622.

**31.** Djoba Siawaya JF, Bapela NB, Ronacher K, Beyers N, van Helden P, Walzl G. Differential expression of interleukin-4 (IL-4) and IL-4 delta 2 mRNA, but not transforming growth factor beta (TGF-beta), TGF-beta RII, Foxp3, gamma interferon, T-bet, or GATA-3 mRNA, in patients with fast and slow responses to antituberculosis treatment. *Clin Vaccine Immunol.* Aug 2008;15(8):1165-1170.

**32.** Sharma N, Saxena S, Agrawal I, et al. Differential Expression Profile of NLRs and AIM2 in Glioma and Implications for NLRP12 in Glioblastoma. *Scientific reports.*9(1):8480.

**33.** Huentelman MJ, Papassotiropoulos A, Craig DW, et al. Calmodulin-binding transcription activator 1 (CAMTA1) alleles predispose human episodic memory performance. *Hum Mol Genet.* Jun 15 2007;16(12):1469-1477.

**34.** Bas-Orth C, Tan YW, Oliveira AM, Bengtson CP, Bading H. The calmodulin-binding transcription activator CAMTA1 is required for long-term memory formation in mice. *Learn Mem.* Jun;23(6):313-321.

**35.** Suzukawa M, Akashi S, Nagai H, et al. Combined Analysis of IFN-Î³, IL-2, IL-5, IL-10, IL-1RA and MCP-1 in QFT Supernatant Is Useful for Distinguishing Active Tuberculosis from Latent Infection. *PLoS ONE.*11(4):e0152483.

**36.** Zheng S, Hedl M, Abraham C. Twist1 and Twist2 Contribute to Cytokine Downregulation following Chronic NOD2 Stimulation of Human Macrophages through the Coordinated Regulation of Transcriptional Repressors and Activators. *The Journal of Immunology.*195(1):217-226.

**37.** Skeldon A, Saleh M. The Inflammasomes: Molecular Effectors of Host Resistance Against Bacterial, Viral, Parasitic, and Fungal Infections. *Frontiers in Microbiology.* 2011-February-17;2(15).

**38.** Moreira-Teixeira Lc, Redford PS, Stavropoulos E, et al. T Cellâ€“Derived IL-10 Impairs Host Resistance to <em>Mycobacterium tuberculosis</em> Infection. *The Journal of Immunology.*199(2):613-623.

**39.** Grenier JM, Wang L, Manji GA, et al. Functional screening of five PYPAF family members identifies PYPAF5 as a novel regulator of NF-ÎºB and caspase-1. *FEBS Letters.* 2002;530(1-3):73-78.

**40.** Ma J, Zhao F, Su W, et al. Zinc finger and interferon-stimulated genes play a vital role in TB-IRIS following HAART in AIDS. *Per Med.* Jul 1;15(4):251-269.

**41.** Vacca M, BÃ¶hme J, Zambetti LP, et al. NLRP10 Enhances CD4(+) T-Cell-Mediated IFNÎ³ Response via Regulation of Dendritic Cell-Derived IL-12 Release. *Frontiers in immunology.*8:1462-1462.

**42.** Eisenbarth SC, Williams A, Colegio OR, et al. NLRP10 is a NOD-like receptor essential to initiate adaptive immunity by dendritic cells. *Nature.*484:510.

**43.** Ellwanger K, Becker E, Kienes I, et al. The NLR family pyrin domainâ€“containing 11 protein contributes to the regulation of inflammatory signaling. *Journal of Biological Chemistry.* February 23, 2018;293(8):2701-2710.

**44.** Ye Z, Lich JD, Moore CB, Duncan JA, Williams KL, Ting JP-Y. ATP Binding by Monarch-1/NLRP12 Is Critical for Its Inhibitory Function. *Molecular and Cellular Biology.* 2008;28(5):1841-1850.

**45.** Franchi L, McDonald C, Kanneganti T-D, Amer A, NÃºÃ±ez G. Nucleotide-Binding Oligomerization Domain-Like Receptors: Intracellular Pattern Recognition Molecules for Pathogen Detection and Host Defense. *The Journal of Immunology.* 2006;177(6):3507-3513.

**46.** Wang L, Manji GA, Grenier JM, et al. PYPAF7, a Novel PYRIN-containing Apaf1-like Protein That Regulates Activation of NF-ÎºB and Caspase-1-dependent Cytokine Processing. *Journal of Biological Chemistry.* August 16, 2002 2002;277(33):29874-29880.

**47.** Hornick EE, Banoth B, Miller AM, et al. Nlrp12 Mediates Adverse Neutrophil Recruitment during Influenza Virus Infection. *The Journal of Immunology.*200(3):1188-1197.

**48.** Okada S, Markle JG, Deenick EK, et al. IMMUNODEFICIENCIES. Impairment of immunity to Candida and Mycobacterium in humans with bi-allelic RORC mutations. *Science (New York, N.Y.).*349(6248):606-613.

**49.** Amelio P, Portevin D, Hella J, et al. HIV Infection Functionally Impairs <span class="named-content genus-species" id="named-content-1">Mycobacterium tuberculosis</span>-Specific CD4 and CD8 T-Cell Responses. *Journal of Virology.*93(5):e01728-01718.

**50.** Chu J-Q, Shi G, Fan Y-M, et al. Production of IL-1Î² and Inflammasome with Up-Regulated Expressions of NOD-Like Receptor Related Genes in Toxoplasma gondii-Infected THP-1 Macrophages. *The Korean journal of parasitology.*54(6):711-717.
